# Distinct Gut Microbiota Profiles in Unruptured and Ruptured Intracranial Aneurysms: Focus on Butyrate-Producing Bacteria

**DOI:** 10.3390/jcm14103488

**Published:** 2025-05-16

**Authors:** Peter Csecsei, Bertalan Takacs, Lídia Pasitka, Reka Varnai, Zoltan Peterfi, Brigitta Orban, Mate Czabajszki, Csaba Olah, Attila Schwarcz

**Affiliations:** 1 Department of Neurosurgery, Medical School, University of Pecs, 7622 Pecs, Hungary; orbanbrigi8@gmail.com (B.O.); schwarcz.attila@pte.hu (A.S.); 2 HCEMM-HUN-REN BRC Mutagenesis and Carcinogenesis Research Group, Institute of Genetics, HUN-REN Biological Research Centre, 6726 Szeged, Hungary; 3 Faculty of Science and Informatics, Doctoral School of Biology, University of Szeged, 6720 Szeged, Hungary; 4 Delta Bio 2000 Ltd., 6726 Szeged, Hungary; 5 Department of Dentistry, Oral and Maxillofacial Surgery, Medical School, University of Pecs, 7622 Pecs, Hungary; lidiamolnar99@gmail.com; 6 Department of Primary Health Care, Medical School, University of Pecs, 7622 Pecs, Hungary; varnai.reka@pte.hu; 7 1st Department of Medicine, Medical School, University of Pecs, 7622 Pecs, Hungary; peterfi.zoltan@pte.hu; 8 Department of Neurosurgery, Borsod-Abaúj-Zemplén County Center Hospital, University Teaching Hospital, 3526 Miskolc, Hungary; czamate@gmail.com (M.C.); olahcs@gmail.com (C.O.)

**Keywords:** aneurysmal subarachnoid hemorrhage, gut microbiome, short-chain fatty acids

## Abstract

**Background:** Gut microbiome composition may influence the risk of intracranial aneurysm rupture. **Methods**: This study analyzed the gut microbiota of 48 patients—24 with ruptured aneurysms (RA) and 24 with unruptured intracranial aneurysms (UIA)—using next-generation sequencing. **Results**: While alpha diversity was similar between groups, beta diversity revealed significant taxonomic differences (Bray–Curtis: *p* = 0.02; unweighted UniFrac: *p* = 0.0291). Both groups were dominated by the phyla *Bacillota*, *Bacteroidota*, and *Proteobacteria*, but genus- and family-level differences were observed. RA patients showed higher abundances of *Anaerotruncus*, *Coprobacillus*, *Sellimonas*, *Hungatella*, and *Ruthenibacterium*, whereas UIA patients exhibited greater levels of *Faecalibacterium*, *Brotolimicola*, *Clostridiaceae*, *Roseburia*, and *Agathobaculum*. Linear discriminant analysis identified one class, 10 genera, and 17 species that differed significantly between groups. Notably, *Faecalibacterium prausnitzii* and *Agathobaculum butyriciproducens*—bacteria known for their anti-inflammatory and neuroprotective properties—were enriched in UIA patients. **Conclusions:** These findings suggest that gut microbiota, particularly short-chain fatty acid–producing bacteria, may contribute to vascular protection and aneurysm pathophysiology. Microbiome-based therapeutic strategies could offer new avenues for the prevention of cerebrovascular disease.

## 1. Introduction

Subarachnoid hemorrhage (SAH) resulting from a ruptured intracranial aneurysm (RA) is a life-threatening neurological emergency associated with high morbidity and mortality [1]. Despite advances in neurocritical care, aneurysmal SAH continues to have poor outcomes in many patients, especially when diagnosis and intervention are delayed [2]. In contrast, unruptured intracranial aneurysms (UIAs) are often discovered incidentally and present a complex clinical dilemma. While some remain stable, others carry a significant risk of rupture. Identifying the biological factors that contribute to aneurysm stability or rupture remains a key focus of neurovascular research. Recent studies have highlighted the gut-brain axis as a significant contributor to the pathophysiology of several neurological diseases, including stroke, Alzheimer’s disease, and Parkinson’s disease [3,4,5,6]. Central to this axis is the gut microbiome—a diverse and dynamic community of microorganisms that influence systemic inflammation, vascular function, and immune modulation through the production of microbial metabolites. Among these metabolites, short-chain fatty acids (SCFAs)—including acetate, propionate, and butyrate—are primarily produced by anaerobic fermentation of dietary fibers by commensal gut bacteria such as *Faecalibacterium prausnitzii*, *Roseburia*, and *Agathobaculum butyriciproducens* [7,8,9,10]. These SCFAs have well-documented anti-inflammatory and neuroprotective effects, including inhibition of NF-κB signaling, enhancement of the blood-brain barrier, and attenuation of neuroinflammation [10,11,12,13,14]. Their presence has also been associated with improved outcomes in cardiovascular and cerebrovascular disorders [15,16]. Conversely, an altered gut microbial profile—often termed dysbiosis—has been implicated in the progression of various inflammatory and vascular diseases. Dysbiosis can result in increased production of pro-inflammatory metabolites and reduced SCFA levels, potentially contributing to vascular wall instability and rupture [17,18,19]. Recent metagenomic analyses have revealed significant differences in gut microbial communities between patients with ischemic stroke and healthy controls, as well as between individuals with symptomatic versus asymptomatic aneurysms [18,20,21]. These findings suggest that the gut microbiome may play a pivotal role in modulating aneurysm behavior and the risk of rupture through inflammation-mediated pathways. Despite this growing body of evidence, little is known about the specific gut microbial signatures associated with ruptured versus unruptured cerebral aneurysms. Understanding the differences in microbiome composition between these groups could provide insight into novel biomarkers for rupture risk and therapeutic targets for aneurysm stabilization.

The aim of the present study is to investigate the gut microbiome composition in patients with ruptured aneurysms (RAs) compared to those with unruptured intracranial aneurysms (UIAs), using next-generation sequencing and metagenomic analysis. By identifying specific bacterial taxa and SCFA-producing species associated with aneurysm stability or rupture, we seek to explore the potential mechanistic link between gut microbiota and cerebrovascular health, and to lay the groundwork for future microbiome-targeted preventive strategies in neurovascular disease.

## 2. Materials and Methods

### 2.1. Study Design and Population

Participants with ruptured aneurysms (RAs) and unruptured intracranial aneurysms (UIAs) were prospectively enrolled between November 2023 and November 2024 at the University of Pécs. The study protocol was approved by the Institutional Review Board (IV/8468-1/2021/EKU and BM/4629-1/2024). Written informed consent was obtained from all participants or their legal representatives prior to inclusion.

Inclusion criteria for the RA group were as follows: 1. age over 18 years; 2. presence of subarachnoid hemorrhage confirmed by non-contrast cranial CT and a source of bleeding (aneurysm) identified via CT angiography and digital subtraction angiography (DSA); and 3. diagnosis established within 24 h of symptom onset (ictus). Exclusion criteria included the following: patients under 18 years of age, non-saccular or traumatic aneurysms (e.g., dural arteriovenous fistula, arterial dissection), known genetic disorders associated with aneurysm formation (e.g., polycystic kidney disease, Ehlers–Danlos syndrome type IV), antibiotic use within one month prior to admission, presence of systemic diseases such as malignancy, hepatic or renal failure, chronic lung disease, inflammatory bowel disease, or any other chronic gastrointestinal disorders, signs of acute or chronic infection at admission, rebleeding events after the initial ictus or clinical deterioration during treatment, and the need for parenteral nutrition before fecal sampling. Inclusion criteria for the UIA group were as follows: 1. age over 18 years; 2. absence of acute or chronic infections; 3. normal laboratory parameters, including renal and liver function tests and inflammatory markers; 4. no history of gastrointestinal or systemic diseases that could impact the microbiome (e.g., inflammatory bowel disease, malignancies); and 5. presence of a stable intracranial aneurysm, unchanged in size and morphology for at least two years since its initial detection.

For all eligible participants, comprehensive clinical data were collected from medical records, including age, sex, cardiovascular risk factors (such as hypertension, diabetes, and smoking status), admission blood pressure, and laboratory parameters. Additional information included baseline neurological assessments using the World Federation of Neurosurgical Societies (WFNS) grading scale and the modified Fisher score. Details of interventions during intensive care—such as mechanical ventilation, placement of an extraventricular or lumbar drain—were also documented. The occurrence and type of infections, the presence of delayed cerebral ischemia (DCI), and clinical outcomes were systematically recorded. All patients underwent follow-up either via telephone interviews or in-person visits. The modified Rankin Scale (mRS) score was assessed at 3 months post-event to evaluate functional outcomes, with a favorable outcome defined as an mRS score of 0 to 3. Patients with unruptured aneurysms who were enrolled in this study received a stool sample collection kit, were instructed on the correct sampling procedure, and personally returned the collected sample. In all cases, we recorded the patient’s dietary habits. In the UIA group, patients’ dietary habits were assessed using a brief questionnaire, which included questions on vegetarian or meat-free diets, food allergies, dairy consumption, meal frequency, and intake of processed foods. For the RA group, this information was obtained either directly from the patients or from their relatives. All participants included in this study (both UIA and RA groups) consumed a mixed diet comprising both animal- and plant-based foods, including dairy products, and none had known food allergies. In the RA group, parenteral nutrition was not administered; balanced calorie-containing enteral nutrition was introduced within 24 h of admission, in accordance with the current guideline [22]. If oral feeding was not possible, nutrition was administered via a nasogastric tube, with an emphasis on transitioning to oral feeding as soon as possible. All patients received nimodipine at a dosage of 60 mg orally, six times per day, starting from the first day to prevent vasospasm. Delayed cerebral ischemia (DCI) was diagnosed based on previously established criteria [23] and only after thoroughly excluding other potential causes. The diagnosis required the agreement of at least two neurointensivists. A patient was considered DCI-positive if clinical deterioration and/or ischemic lesions confirmed by CT/MR occurred within six weeks of admission and could not be attributed to other causes. Large vessel vasospasm, in the absence of clinical symptoms or a new ischemic lesion, was not classified as DCI [23].

Systemic and central nervous system infections were defined by the presence of infection symptoms along with fever (>38 °C), elevated C-reactive protein (a rising CRP level following an initial peak), and/or procalcitonin levels exceeding 0.5 ng/mL. Diagnosis was further supported by positive test results, such as chest X-ray findings, cerebrospinal fluid (CSF) analysis, blood cultures, or urine analysis.

### 2.2. Fecal Sample Collection

The stool samples in both groups were collected using a Stool Sample Collection & Stabilization Kit (Canvax Biotech, Valladolid, ES, Spain), following the manufacturer’s instructions. The samples from patients in the RA group were stored frozen at −20 °C until analysis. Patients in the UIA group were provided with stool collection containers and instructed on the proper procedure for collecting samples at home using the same Canvax Stool Sample Collection & Stabilization Kit (Canvax Biotech, Valladolid, ES, Spain). The instructions included guidelines on how to collect the stool sample, ensuring minimal contamination, and how to properly seal the collection container. After collection, patients were advised to immediately store the samples at −20 °C to preserve the integrity of the specimen. The frozen samples were then required to be brought back to the clinic as soon as possible for further analysis. In the RA group, stool samples were collected either by the patient or by a specialized assistant with the help of healthcare professionals, following the guidelines for sample collection. The stool sample collection took place 72 h after hospital admission to minimize the effects of hospitalization. In all cases, sampling was conducted before the use of antibiotics or the onset of infection.

### 2.3. DNA Isolation

Sample collection and DNA isolation were performed using the QIAamp PowerFecal Pro DNA Kit. Next-generation sequencing libraries were prepared using Illumina Nextera XT DNA Library Preparation Kit (FC-131-1096 Illumina, San Diego, CA, USA) according to the manufacturer’s instructions. For quality control, libraries were run on a BioAnalyzer2100 instrument using the High Sensitivity DNA Kit (5067-4626 Agilent, Santa Clara, CA, USA). Fragment libraries were sequenced on an Illumina NextSeq2000 instrument with 2 × 150 bp chemistry (20024904 Illumina).

### 2.4. Metagenomic Analysis

NGS data were preprocessed with Trimmomatic [24] and Cutadapt [25], using the default parameters and quality-checked with FastQC [26] and MultiQC [27]. Bacterial composition of the samples was identified by running MetaPhlAn4 [28], with the mpa_vJun23_CHOCOPhlAnSGB_202403 version of the CHOCOPhlAn database. MetaPhlAn4 identified several taxa that lack official Latin names and are instead represented by alphanumeric identifiers. These taxa, referred to as genome bins, are predicted groups derived from metagenomic sequences that could not be classified into known species. MetaPhlAn4 can detect these predicted taxa at sub-species levels, but they often provide limited additional information beyond the species level. Each predicted taxon has a unique identifier assigned by the database authors, where the first letter denotes the taxonomic level (e.g., SGB for species-level genome bins, GGB for genus-level genome bins). Bar charts were used to depict the most abundant taxa at each taxonomic level, with remaining taxa grouped under the “Other” category. Sample composition was visualized in Python 3.13.3, using the matplotlib [29] and seaborn [30] packages. Diversity metrics were calculated in R, using the microbiome [31], rbiom [32] and ape [33] packages. For calculating weighted and unweighted UniFrac distances, the mpa_vJun23_CHOCOPhlAnSGB_202403.nwk taxonomic tree, included with MetaPhlan4, was used. PCoA analysis, statistical testing and visualization of beta diversity were conducted in R, using the phyloseq [34] and ggplot2 [35] packages. Differences between groups were assessed using an ANOVA test, with the resulting *p*-values displayed on the plots. The ellipses represent 95% confidence intervals. Differential abundance analysis was conducted using LEfSe [36]. In the LEfSe results, green represents taxa overrepresented in the UIA group, while red represents taxa overrepresented in the RA/SAV group. The x-axis displays the linear discriminant analysis (LDA) effect size, where higher values indicate stronger differentiation between groups. Heatmaps were generated for taxa identified as significant by LEfSe, maintaining the same order as in the LEfSe bar plots. Color intensity on the heatmaps corresponds to the relative abundance of each taxon, with values log-transformed (base 10) for visualization.

## 3. Results

### 3.1. Patient Characteristics

Forty-eight subjects (24 with RA and 24 patients with UIAs) met the eligibility criteria and completed the entire study protocol. Sixteen (67%) were women in the RA group and 17 (71%) in the UIA group (*p* > 0.05). The average age of the population (RA + UIA) was 61 ± 12 years. No significant difference was observed between the two groups in the frequency of comorbidities such as hypertension, diabetes, or IHD. There was also no significant difference between the two groups in terms of aneurysm size or localization. A total of 24 patients with UIAs were followed up for 65 ± 90 months (interquartile range, 26–55 months). Table 1 presents the detailed characteristics of the patient population.

### 3.2. Gut Microbiome Analysis in the RA and UIA Groups

A total of 1296 OTUs (Operational Taxonomic Units) were obtained, using Venn diagrams to visually show the number of shared and unique OTUs in different groups. The results showed that there were about 709 OTUs shared in the two groups, 292 and 295 OTUs in the UIA group and the RA group, respectively.

**Figure 1:**
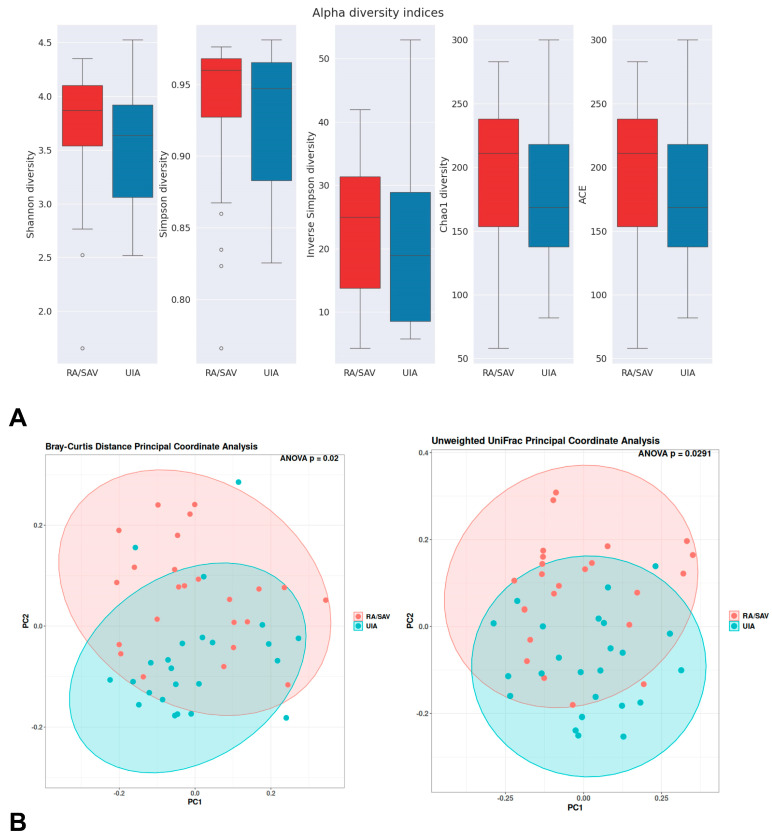
(**A**) Boxplots showing alpha diversity metrics (Shannon diversity, Simpson diversity, Inverse Simpson diversity, Chao1, and ACE) for the RA/SAV (red) and UIA (blue) groups. Statistical significance was assessed using ANOVA. (**B**) Principal Coordinate Analysis (PCoA) plots based on Bray–Curtis (left) and unweighted UniFrac (right) distance metrics. The Bray–Curtis analysis reveals a significant difference in microbial composition between groups (ANOVA *p* = 0.02), while the unweighted UniFrac analysis indicates significant variation in microbial presence/absence (ANOVA *p* = 0.0291). Color code: RA/SAV group = red, UIA group = blue. Sample sizes: RA/SAV, *n* = 24; UIA, *n* = 24. RA/SAV: patients with ruptured aneurysm; UIA: patients with unruptured aneurysm.

Between the RA and UIA groups, no difference was observed for alpha diversity, which includes species richness (represented by Chao1) and richness and evenness (represented by Shannon index, Simpson index, and inverse Simpson index) of the microbial community, Figure 1A.

To assess variation in microbial composition, we performed principal coordinates analysis based on Bray–Curtis dissimilarity and unweighted UniFrac distances. The b-diversity analysis demonstrated significant differences in gut taxonomic composition between patients with ruptured aneurysm and UIA patients by both methods (Bray–Curtis: *p* = 0.02, unweighted UniFrac: *p* = 0.0291, Figure 1B).

**Figure 2:**
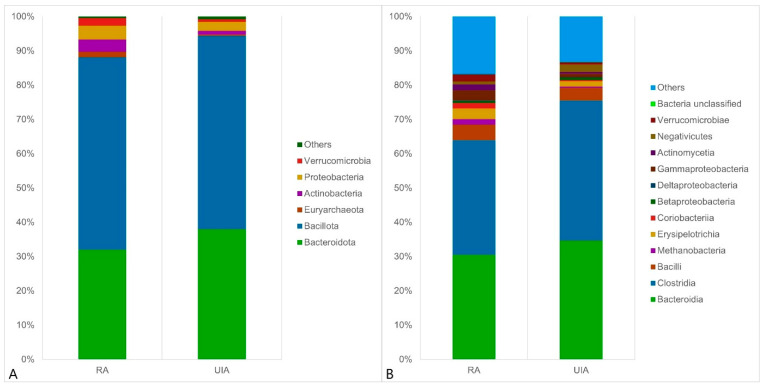

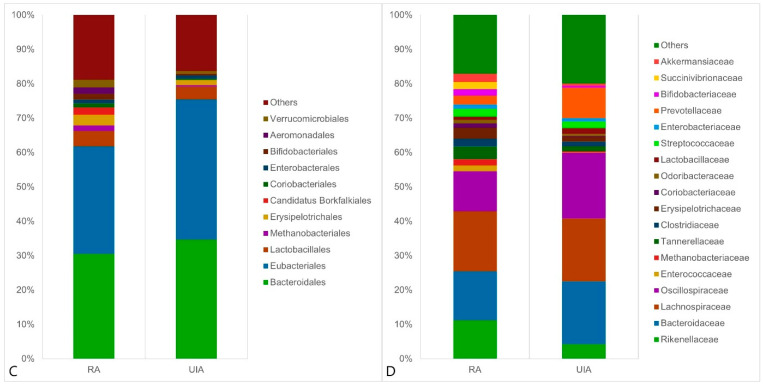
Stacked bar plots showing the relative abundance of gut microbiota at four taxonomic levels: (**A**) phylum, (**B**) class, (**C**) order, and (**D**) family. Color codes represent individual microbial taxa (legend provided in the figure). Sample sizes: RA/SAV, *n* = 24; UIA, *n* = 24. RA: patients with ruptured aneurysm; UIA: patients with unruptured aneurysm.

Next, the composition of the gut microbiome at the kingdom, phylum and family levels in two groups was evaluated. The overall microbial compositions of RA and UIA groups were examined at different taxonomic levels. At the phylum level, *Bacillota*, *Bacteroidota*, *Proteobacteria*, *Actinobacteria,* and *Verrucomicrobia* were the dominant bacteria, Figure 2A. A mild decrease was observed in the abundance of *Euryarchaeota* among UIA patients, whereas *Actinobacteria* were slightly more abundant in the RA group. At the class level, *Bacteroidia*, *Clostridia*, *Bacilli*, *Erysipelotrichia,* and *Gammaproteobacteria* were the dominant bacteria, Figure 2B. We found that the relative abundance of *Coriobacteriia* increased in the RA group compared with the UIA group. At the order level, *Bacteroidales*, *Eubacteriales*, *Lactobacillales*, and *Verrucomicrobiales* were the dominant bacteria, Figure 2C. The relative abundance of *Lactobacillales, Methanobacteriales*, *Erysipelotrichales,* and *Coriobacteriales* increased in the RA group compared to the UIA group. At the family level, *Rikenellaceae*, *Bacteroidaceae*, *Lachnospiraceae*, *Oscillospiraceae*, *Erysipelotrichaceae,* and *Prevotellaceae* were the dominant bacteria, Figure 2D. In RA patients, the relative abundance of *Rikenellaceae*, *Enterococcaceae*, *Methanobacteriaceae*, *Tannerellaceae*, *Erysipelotrichaceae,* and *Coriobacteriaceae* increased, while that of *Oscillospiraceae* and *Lactobacillaceae* decreased significantly compared with the UIA group.

**Figure 3:**
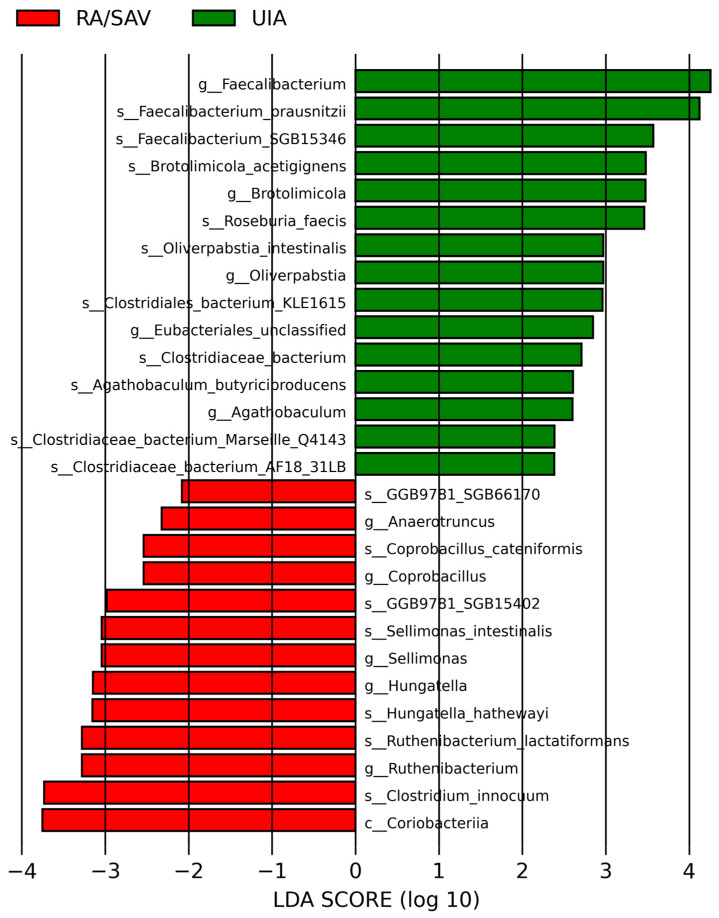
LEfSe (linear discriminant analysis effect size) results showing microbial taxa with significant differential abundance between RA/SAV and UIA groups. The x-axis indicates LDA scores (log_10_), reflecting the strength of association with each group. Color code: taxa enriched in RA/SAV = red; taxa enriched in UIA = green. Sample sizes: RA/SAV, *n* = 24; UIA, *n* = 24. RA/SAV: patients with ruptured aneurysm; UIA: patients with unruptured intracranial aneurysm.

In the linear discriminant analysis effect size analysis, we identified one class, 10 genera, and 17 species that were significantly different in their relative abundance in the RA and UIA groups, Figure 3.

**Figure 4:**
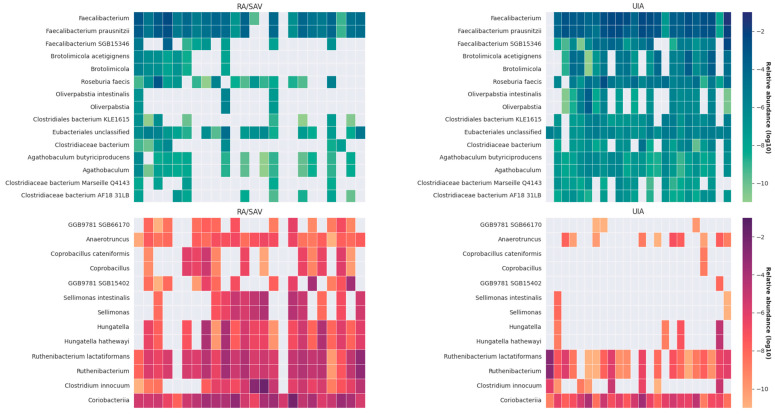
Heatmaps displaying the log_10_-transformed relative abundance of selected microbial taxa in RA and UIA groups. Top heatmaps: taxa distribution in RA (**left**) and UIA (**right**) using a blue–green color scale. Bottom heatmaps: additional taxa distribution in RA (**left**) and UIA (**right**) using a red–purple color scale. Color intensity corresponds to relative abundance: darker colors = higher abundance, lighter colors = lower abundance. Sample sizes: RA/SAV, *n* = 24; UIA, *n* = 24. RA/SAV: patients with ruptured aneurysm; UIA: patients with unruptured aneurysm.

The LDA distribution diagram analysis (LDA score > 2.0, *p* < 0.001) showed a clear alteration of the microbiome characterized by higher *Faecalibacterium*, *Brotolimicola*, *Clostridiaeceae*, *Eubacteriales*, *Roseburia* and *Agathobaculum* levels in UIA patients. However, *Anaerotruncus*, *Coprobacillus*, *Sellimonas*, *Hungatella* and *Ruthenibacterium* levels were significantly more abundant in RA patients. Heatmap of the discriminative taxa revealed by LEfSe. Most of the discriminative taxa in the RA group did not exist in the UIA group, Figure 4.

## 4. Discussion

This study provides novel insights into the role of gut microbiota composition in the pathophysiology of intracranial aneurysms (IAs), with a specific focus on differences between ruptured aneurysms (RAs) and unruptured intracranial aneurysms (UIAs). Our findings indicate that gut microbial profiles are distinctly different between these groups and may reflect mechanisms influencing aneurysm stability and rupture risk.

### 4.1. Gut Microbiota in Intracranial Aneurysm (IA) Patients

Analysis revealed significantly higher levels of SCFA-producing bacteria, including *Faecalibacterium prausnitzii*, *Roseburia faecis*, *Agathobaculum butyriciproducens*, *Clostridiaeceae*, and *Brotolimicola*, in UIA patients. These bacteria are well-known for producing butyrate, a short-chain fatty acid (SCFA) with documented anti-inflammatory, neuroprotective, and endothelial-protective properties [7,8,9,10,14]. Butyrate enhances gut barrier integrity and modulates systemic and central nervous system (CNS) inflammation via inhibition of the NF-κB pathway [10,11,12,13]. Their abundance in UIA patients suggests that such microbes may contribute to aneurysm stability.

*Agathobaculum butyriciproducens* (SR79), in particular, has been shown to reduce dopaminergic neuronal death and glial activation in Parkinson’s disease models and to enhance cognitive function and reduce inflammation in LPS-induced mouse models [9,37]. Agathobacter species, which are closely related and also butyrate producers, have been found to be downregulated following traumatic brain injury (TBI) in both human and animal models [38,39].

Likewise, *Faecalibacterium prausnitzii* exerts anti-inflammatory effects via inhibition of NF-κB and promotes mucosal healing in colitis models [10,11,12,13,14]. Its reduced abundance has been consistently associated with inflammatory bowel disease, Alzheimer’s disease (AD), and several psychiatric disorders [3,4]. The observed preservation of this genus in UIA patients may reflect better vascular and neuroimmune regulation.

In contrast, RA patients were enriched with potentially pathogenic or dysbiosis-associated genera such as Anaerotruncus, *Coprobacillus*, *Sellimonas*, *Hungatella*, and *Ruthenibacterium*. These taxa have been linked to inflammation and poorer neurological or cardiovascular outcomes. For instance, *Sellimonas* intestinalis was correlated with greater stroke severity and worse functional outcomes in acute ischemic stroke (AIS) [40], and also linked to myocardial infarction risk [41]. *Hungatella* abundance has been elevated in hemorrhagic stroke and is associated with cerebrovascular damage [42,43].

Sun et al. [18] found *Ruthenibacterium*, *Anaerotruncus*, and *Clostridiales* order members to be more abundant in symptomatic UIA patients, which aligns with our finding of their enrichment in RA cases. These taxa are involved in metabolic pathways like peptidoglycan biosynthesis, which promotes inflammation, and decreased propionate metabolism, which has immune-modulatory effects [7].

Campylobacter, particularly *Campylobacter ureolyticus*, was previously found in higher abundance in RA patients in other studies [20]. However, in our cohort, its abundance was minimal and showed no significant group difference. This discrepancy may be explained by regional variations in diet, hygiene, and environmental exposures [19].

Zhu et al. [15] reported higher levels of *Faecalibacterium* and *Roseburia* in stroke patients with favorable outcomes (mRS 0–2), reinforcing our observation that these genera may contribute to vascular resilience. Xu et al. [21] also confirmed greater abundance of *Faecalibacterium*, *Agathobaculum*, and *Roseburia* in UIA and control subjects compared to RA cases. Luo et al. [16] showed that *Faecalibacterium prausnitzii* was negatively correlated with stroke severity and inflammatory cytokines in AIS.

Finally, our data demonstrated an altered Firmicutes/Bacteroidota ratio in RA patients, a dysbiosis marker previously associated with metabolic endotoxemia and cardiovascular risk [19,20].

### 4.2. Gut Microbiota in Other Intracranial and Neurological Conditions

Beyond IAs, numerous neurological diseases share common gut microbiota alterations. In Alzheimer’s and Parkinson’s disease, reductions in Faecalibacterium and other SCFA producers have been observed [5,6]. *Eubacterium hallii* and *Eubacterium rectale*, also depleted in neurodegenerative diseases, support the notion that loss of anti-inflammatory commensals may promote neurovascular vulnerability [3].

Psychiatric disorders such as schizophrenia, depression, and bipolar disorder also show reduced *Faecalibacterium* levels, indicating shared inflammatory or metabolic disruptions involving the gut-brain axis [4].

The downregulation of Agathobacter in TBI and animal brain injury models [38,39] underscores a broader protective role for butyrate-producing taxa. These changes, though studied in other CNS pathologies, parallel the microbial shifts we observed between stable and ruptured aneurysm cases.

### 4.3. Mechanistic Implications and Future Directions

Microbial dysbiosis in RA patients may contribute to aneurysm rupture through multiple mechanisms: increased gut permeability, systemic endotoxemia, and activation of pro-inflammatory pathways. Bacteria like *Coprobacillus* and *Hungatella* could exacerbate vascular inflammation and remodeling, while SCFA producers help preserve endothelial and immune balance [17,18,19].

These observations support the idea that gut microbiota alterations are not merely epiphenomena but may directly influence cerebrovascular outcomes. The identification of taxa with protective or pathogenic potential offers new directions for biomarker development and therapeutic targeting.

### 4.4. Limitations

Several important limitations must be acknowledged, and they significantly affect the interpretation and generalizability of our findings. First and foremost, the relatively small sample size (*n* = 48), although consistent with other exploratory microbiome studies, inherently limits the statistical power of our analyses. This sample size increases the risk of both Type I and Type II errors and makes it difficult to confidently extrapolate results to broader patient populations. The findings should, therefore, be considered preliminary and hypothesis-generating rather than conclusive.

Second, the two study cohorts—patients with ruptured aneurysms (RA) and those with unruptured intracranial aneurysms (UIA)—were enrolled under markedly different clinical conditions. RA patients were acutely ill, hospitalized, and often required intensive medical interventions, whereas UIA patients were generally stable and assessed in an outpatient setting. These differences introduce substantial confounding variables, including but not limited to physiological stress, mechanical ventilation, sedation, and enteral feeding, all of which are known to influence gut microbiota composition [17,44]. Thus, the differences observed between groups may partly reflect acute illness effects rather than pathophysiological features specific to aneurysm rupture.

Third, the cross-sectional design of this study restricts causal inference. It is not possible to determine whether the observed microbiome differences contribute to aneurysm rupture or are instead a result of the rupture event and its associated systemic inflammatory response. Longitudinal designs will be necessary to assess the temporal relationship between microbiota changes and aneurysm progression or rupture risk.

Furthermore, while stool samples from RA patients were collected within 72 h of admission and before antibiotic exposure, the acute hospitalization phase inherently involves confounders such as altered diet, stress responses, and supportive therapies, which can independently impact microbiome profiles [17,44]. Several ICU-related interventions in RA patients may have influenced gut microbiota independently of aneurysm pathophysiology. Even brief fasting or nutritional deprivation can reduce microbial diversity and promote dysbiosis [45]. Proton pump inhibitors (PPIs) may alter gastric pH and allow overgrowth of non-native species [46], while critical illness and sedation can impair gut barrier function and microbial balance [47]. Although samples were collected before antibiotic use, early intensive care measures likely introduced bias. The lack of detailed records on medications and nutrition further limits our ability to separate disease-driven from treatment-related microbiota changes. Although we attempted to minimize these effects, their influence cannot be fully excluded.

Additional biases stem from the absence of systematically collected dietary and medication data. These are both well-established modulators of the gut microbiome and could account for some of the variability between groups [19]. Similarly, the lack of functional metagenomic or metabolomic profiling prevents direct assessment of microbial metabolic pathways such as short-chain fatty acid (SCFA) production, which is central to our interpretative framework [48,49]. Our findings remain descriptive and compositional; the functional relevance of the identified taxa remains to be validated.

Finally, the study population was geographically, ethnically, and culturally homogeneous, drawn entirely from a single center in Hungary. This may limit generalizability, as gut microbiota composition is known to vary across populations due to differences in genetics, environment, and diet [19,20].

In light of these limitations, we underscore that the present results must be interpreted with caution. While they support the hypothesis that gut microbiota may play a role in the stability and rupture of intracranial aneurysms, the conclusions remain tentative. Robust validation in larger, multicenter, and functionally annotated cohorts is essential for confirming these preliminary findings.

## 5. Conclusions

Our study demonstrates distinct gut microbiota profiles between patients with ruptured and unruptured intracranial aneurysms. Enrichment of SCFA-producing genera in UIA patients may reflect protective, anti-inflammatory effects that contribute to vascular stability. Conversely, RA patients exhibited an increased abundance of potentially pathogenic, pro-inflammatory taxa. These findings support the emerging concept that the gut microbiome plays a role in cerebrovascular disease, including aneurysm rupture risk. Future work should aim to validate these associations in larger, longitudinal studies and explore the potential of microbiota-targeted interventions in aneurysm management.

## Figures and Tables

**Table 1:** Detailed characteristics of study population.

Variable	RA (*n* = 24)	UIA (*n* = 24)	*p*-Value
Age (mean ± SD)	59 ± 14	63 ± 10	0.39
Female, N (%)	16 (67)	17 (71)	0.5
Hypertension, N (%)	15 (63)	20 (83)	0.09
Diabetes, N (%)	3 (13)	7 (29)	0.14
IHD, N (%)	12 (50)	6 (25)	0.07
Smoking, N (%)	14 (58)	10 (42)	0.19
WFNS, median (IQR)	1.5 (1–4)	N/A	N/A
Fisher score, median (IQR)	3 (1.5–4)	N/A	N/A
Loss of consciousness during ictus, N (%)	14 (58)	N/A	N/A
Aneurysm location			0.36
anterior circulation	18 (75)	20 (83)	
posterior circulation	6 (25)	4 (17)	
Size of aneurysm, mm	6 (5–8)	6 (5–10)	0.76
Lumbal drain, N (%)	18 (75)	N/A	N/A
Mechanical ventilation, N (%)	8 (33)	N/A	N/A
Decompressive craniotomy, N (%)	1 (4)	N/A	N/A
Extraventricular drainage, N (%)	3 (13)	N/A	N/A
Delayed cerebral ischemia, N (%)	3 (13)	N/A	N/A
Infection, N (%)	9 (38)	N/A	N/A
3-month mRS score	2 (1–3)	N/A	N/A

RA, ruptured aneurysm; UIA, unruptured intracranial aneurysm; SD, standard deviation; N, number; IHD, ischemic heart disease; WFNS, World Federation of Neurological Societies score; IQR, interquartile range; mRS score, modified Rankin score. N/A: not applicable.

## Data Availability

The datasets used and/or analysed during the current study are available from the corresponding author on reasonable request.

## Abbreviations

The following abbreviations are used in this manuscript:

6-OHDA	6-Hydroxydopamine
AD	Alzheimer’s Disease
AIS	Acute Ischemic Stroke
ANOVA	Analysis of Variance
aSAH	Aneurysmal Subarachnoid Hemorrhage
CRP	C-Reactive Protein
CSF	Cerebrospinal Fluid
DCI	Delayed Cerebral Ischemia
DNBS	Deoxynivalenol-Sulfate
DSS	Dextran Sodium Sulfate
IBD	Inflammatory Bowel Disease
IGF-1	Insulin-like Growth Factor-1
IHD	Ischemic Heart Disease
IQR	Interquartile Range
LDA	Linear Discriminant Analysis
LEfSe	Linear Discriminant Analysis Effect Size
LPS	Lipopolysaccharide
log10	Logarithm Base 10
MetaPhlAn	Metagenomic Phylogenetic Analysis
mFS	Modified Fisher Score
NGS	Next-Generation Sequencing
NF-κB	Nuclear Factor Kappa B
OTUs	Operational Taxonomic Units
PCoA	Principal Coordinate Analysis
RA	Ruptured Aneurysm
SAH	Subarachnoid Hemorrhage
SCFAs	Short-Chain Fatty Acids
UIA	Unruptured Intracranial Aneurysm
WFNS	World Federation of Neurological Societies Score
